# Effect of eNOS on Ischemic Postconditioning-Induced Autophagy against Ischemia/Reperfusion Injury in Mice

**DOI:** 10.1155/2019/5201014

**Published:** 2019-02-10

**Authors:** Jun Shao, Chen Miao, Zhi Geng, Maohong Gu, Yanhu Wu, Qingguo Li

**Affiliations:** ^1^Department of Cardiothoracic Surgery, The Second Affiliated Hospital of Nanjing Medical University, Nanjing, Jiangsu 210011, China; ^2^Department of Pathology, The First Affiliated Hospital of Nanjing Medical University, Nanjing, Jiangsu 210029, China; ^3^Department of Obstetrics and Gynecology, The Affiliated Nanjing First Hospital to Nanjing Medical University, Nanjing, Jiangsu 210006, China; ^4^Department of Cardiothoracic Surgery, The First Affiliated Hospital of Nanjing Medical University, Nanjing, Jiangsu 210029, China

## Abstract

Autophagy is involved in the development of numerous illnesses, including ischemia/reperfusion (I/R). Endothelial nitric oxide synthase (eNOS) participates in the protective effects of ischemic postconditioning (IPostC). However, it remains unclear whether eNOS-mediated autophagy serves as a critical role in IPostC in the hearts of mice, in protecting against I/R injury. In the present study, the hearts of mice with left anterior descending coronary artery ligation were studied as I/R models. H9c2 cells underwent exposure to hypoxia/reoxygenation (H/R) and were examined as* in vitro* model. IPostC reduced mice myocardial infarct size and improved the structure of the heart. IPostC increased the formation of autophagosomes and increased the phosphorylation of eNOS and adenosine monophosphate-activated protein kinase (AMPK). Autophagy and eNOS inhibition suppressed the cardioprotective effects of IPostC. AMPK or eNOS inhibition abolished the improvement effect of IPostC on autophagy. AMPK inhibition decreased eNOS phosphorylation in the heart. Additionally, H9c2 cells suffering hypoxia were used as in vitro model. Autophagy or eNOS inhibition abolished the protective effects of hypoxic postconditioning (HPostC) against H/R injury. AMPK and eNOS inhibition/knockout decreased autophagic activity in the HPostC group. These results indicated that IPostC protects the heart against I/R injury, partially via promoting AMPK/eNOS-mediated autophagy.

## 1. Introduction

Ischemic heart disease is a serious health problem worldwide [1]. Ischemia/reperfusion (I/R) injury often occurs in myocardial infarction therapy, which reduces the therapeutic effects and aggravates myocardial injury [2]. Therefore, it is imperative to identify a therapeutic strategy for I/R injury.

As early as 2003, ischemic postconditioning (IPostC) showed obvious myocardial protective effect in an animal model, markedly reducing infarct size compared with controls [3]. In 2005, the first clinical study demonstrated that IPostC could significantly reduce myocardial necrosis in STEMI patients [4]. Numerous studies in recent years have confirmed that ischemic postconditioning has a protective effect on hearts with I/R [5–7], with studies primarily focusing on mitochondrial injury and oxidative stress [8, 9], such as through blocking the mitochondrial permeability transition pore, activating ATP-dependent potassium channels in mitochondria and improving endothelial functions [10]. Other important mechanisms may also contribute to IPostC; however, these have not been completely identified and elucidated.

Previous studies have reported that autophagy participates in the pathological progress of I/R injured heart [11, 12]. Autophagy is a cellular, physiological process that mediates the degradation of unnecessary or damaged organelles and proteins [13]. A baseline level of autophagy is required for maintaining essential cardiac function due to its critical role in controlling the quality of proteins and organelles [14]. Deregulating the genes closely associated with autophagy may result in cardiac disorders [11]. In an I/R injured heart, autophagy is activated, and partly functions to remove cytotoxic ubiquitinated proteins and attenuate protein aggregation in the myocardium. The role of autophagy in a heart with I/R injury has become a potential therapeutic interest.

AMP-activated protein kinase (AMPK) is activated under the condition of changes in cellular energy levels. Study shows that AMPK activation protects diabetic heart against ischemia-reperfusion injury and also serves an important role in the protective effect of IPostC [15]. IPostC attenuates I/R injury via increasing the phosphorylation of AMPK and endothelial nitric oxide synthase (eNOS) in H9c2 cells* in vitro [16]*. IPostC also decreases the cardiac infarction size in an I/R injured heart via the AMPK-mediated signaling pathway [17]. Additionally, a previous study demonstrated that the first orally active adiponectin receptor activator AdipoRon increased autophagy via AMPK-mediated signaling, in order to protect the heart against I/R [18]. The cell-permeable compound D942 also activates AMPK and protects the heart against I/R injury via inducing autophagy [19]. Therefore, autophagy induced by AMPK may be involved in the protective effect of IPostC.

eNOS is an important source of nitrogen oxide (NO) and participates in the regulation of physiological and pathological functions in the cardiovascular system [20]. Numerous studies have demonstrated that AMPK activation could significantly increase the phosphorylation of eNOS in different organs and tissues [21–23]. A previous study reported that the expression of eNOS is increased by IPostC against I/R injury in H9c2 cells [24]. In addition, in a renal I/R injury model in rats, caloric restriction was demonstrated to increase the expression of eNOS, sirtuin 1 (SIRT1), peroxisome proliferator-activated receptor gamma coactivator 1*α* (PGC-1*α*), and autophagy was increased, in order to protect against the I/R injury [25]. These studies indicate that eNOS may be involved in autophagy and participate in IPostC.

At present, to the best of our knowledge, there has been no specific study regarding the role of eNOS in the cardioprotective effect of IPostC. In the current study, it was hypothesized that IPostC protects the heart against I/R injury via promoting AMPK/eNOS-mediated autophagy.

## 2. Materials and Methods

### 2.1. Ethics Statement

Animal experiments were approved by Nanjing Medical University Animal Care and Use Committee. Animal experiments procedures were carried out strictly according to the regulations of the ethics committee of the International Association for the Study of Pain and the Guide for the Care and Use of Laboratory Animals (The Ministry of Science and Technology of China, 2006).

### 2.2. Reagents and Antibodies

eNOS (D9A5L) Rabbit mAb (#32027), p-eNOS^Ser1177^ (C9C3) Rabbit mAb (#9570), AMPK*α* (D5A2) Rabbit mAb (#5831), p-AMPK^Thr172^ (D4D6D) Rabbit mAb (#50081), LC3A/B Antibody (#4108), SQSTM1/p62 (D1Q5S) Rabbit mAb (#39749), Anti-rabbit IgG, HRP-linked Antibody (7074), and Anti-mouse IgG, HRP-linked Antibody (7076) antibodies were purchased from Cell Signaling Technology, Inc. (Danvers, MA, USA). The autophagy inhibitor 3-Methyladenine (3-MA) (M9281), eNOS inhibitor (L-NIO) (I134), AMPK inhibitor (Compound C) (171260), and GAPDH rabbit antibody (HPA040067) were purchased from Sigma-Aldrich (Merck KGaA, Darmstadt, Germany). Dulbecco's modified Eagle's medium (DMEM) (21885108) and fetal bovine serum (FBS) (10437028) were purchased from Gibco (Thermo Fisher Scientific, Inc., Waltham, MA, USA).

### 2.3. Ischemia/Reperfusion Model Establishment and Infarct Size Measurement

Adult male C57/B6 mice (weight 25-30 g) were anesthetized with 4% chloral hydrate (100 mg/kg, i.p.) [26]. Control group: a left lateral thoracotomy and pericardiectomy without ligating the left anterior descending coronary artery were perform to mice. Mice I/R heart model was established as follows: heart ischemia for 30 min and reperfusion for 60 min. The left anterior descending coronary artery was ligated for 30 min using an 8-0 nylon suture and two cotton coils were placed under the suture to prevent arterial injury following a left lateral thoracotomy and pericardiectomy. IPostC (30 sec of reperfusion and 30 sec of ischemia for three cycles) was performed at the first 3 minutes of reperfusion, followed by an additional 60 min reperfusion [26]. Mice were administered with 3-MA (15mg/kg, i.p.) and eNOS inhibitor L-NIO (30 mg/kg; i.p.) 30 min prior to reperfusion. An elevated ST segment on the electrocardiogram was considered to indicate regional ischemia.

Hearts were collected and sliced into 2-mm transverse sections from apex to base. Slices were then incubated for 15 min with 1% triphenyltetrazolium chloride (Sigma-Aldrich; Merck KGaA) in phosphate-buffered saline (PBS) at 37°C. Data analysis was performed with Image-Pro Plus software version 6.0.

### 2.4. Hematoxylin and Eosin (H&E) Staining

Researchers collected the mouse hearts, fixed with 10% formalin for 24 h and embedded in paraffin, and subsequently cut the heart to microtome sections (4-*μ*m thick), and stained the sections with H&E. Images were obtained with a Zeiss microscope using the 10× objective.

### 2.5. TUNEL Staining

Hearts were fixed in 10% formalin and embedded in paraffin for TUNEL staining (Roche). In brief, using a 40× objective, images were taken with a Leica DM 2500 microscope (Leica).

### 2.6. H9c2 Cells Culture

The H9c2 cell line was purchased from the Cell Bank of Type Culture Collection of the Chinese Academy of Sciences (Shanghai, China), cultured in DMEM (Life Technologies; Thermo Fisher Scientific, Inc.) containing 10% FBS. Cells were grown to 80% confluence in 75 cm^2^ flasks (37°C, 5% CO_2_) in preparation for experiments. The cells were made quiescent by serum starvation for 24 h. Glucose and serum-free DMEM saturated with N_2_ gas for 1 h was used after cells were moved to the hypoxia container. The experimental protocols are as follows: the control group and H9c2 cells were incubated in serum-free, low-glucose DMEM in a normoxic incubator. Cells in the hypoxia/reoxygenation (H/R) group were subjected to hypoxia for 1 h [27] and reoxygenation for 1 h. Cells in the hypoxic postconditioning (HPostC) group were subjected to HPostC including three cycles of hypoxia (5 min) and reoxygenation (5 min) following hypoxia for 1 h, followed by 30 min of reoxygenation [3, 28]. During 5 min of hypoxia, we are continuously feeding nitrogen into the culture bottle to replace the air rapidly using a tube. During 5 min of reoxygenation, we are continuously feeding gas mixture (95% air and 5% CO_2_) into the culture bottle to replace nitrogen rapidly using a tube (3). The AMPK inhibitor Compound C (5 *μ*mol/l), eNOS inhibitor L-NIO (10 *μ*mol/l), and autophagy inhibitor 3-MA (5 mM) were purchased from Sigma-Aldrich (Merck KGaA) and administered from the beginning of hypoxic postconditioning.

### 2.7. SiRNA Transfection in H9C2 Cells

Lipofectamine reagent was used for eNOS siRNA (purchased from Santa Cruz Biotechnology) and mock control oligonucleotides transfecting according to the manufacturer's instructions. siRNAs transfection concentration was 100 nM. Transfected cells were then incubated in new culture media for an additional treatment as indicated.

### 2.8. Cell Viability Assessment

CCK-8 assay (Dojindo Molecular Technologies, Inc., Kumamoto, Japan) was used to measure cell viability. CCK-8 solution (200 *μ*l) was added to each well of a 96-well plate with H9c2 cells (2.0x10^4^ cell/well) 1 h at 37°C and was subsequently incubated for 1 h at 37°C. The optical density (OD) of each well at 450 nm was recorded using a Microplate Reader (Thermo Fisher Scientific, Inc.). The cell viability (% of control) was analyzed using the equation: Cell viability (%) = (OD_test_ - OD_blank_)/(OD_control_ - OD_blank_) x 100%, where OD_control_ is the optical density of the control sample and OD_blank_ is the optical density of the wells without H9c2 cells.

### 2.9. Western Blotting

RIPA lysis buffer [50 mM Tris-HCl (pH=7.5), 150 mM NaCl, 1% NP-40 (vol/vol), 1 mg/ml SDS, 5 mg/ml hyodeoxycholic acid sodium, 1 mM PMSF, 10 mg/ml aprotinin, 10 mg/ml leupeptin, and 10 mg/ml pepstatin A] was added to the heart tissue and homogenized using IKA homogenizer for 30 min on ice. H9c2 cells were lysed using RIPA buffer for 30 min on ice. The cell suspension was centrifuged at 12,000 x g for 15 min. Bicinchoninic Acid Protein assay kit (Beyotime Institute of Biotechnology, Haimen, China) was used to measure the concentrations of the lysates. Total protein (30 *μ*g) was separated using 8-15% SDS polyacrylamide gels and were transferred onto polyvinylidene difluoride membranes. The membranes were incubated with primary antibodies at 4°C overnight after blocking with 5% fat-free milk 2 h at room temperature. The membranes were washed with TBST 3 times and incubated with secondary antibodies for 2 h at room temperature. Following washing with TBST for 3 times, the membranes were detected using enhanced chemiluminescence (Pierce; Thermo Fisher Scientific, Inc.) and quantified by Kodak Image Station 4000 MM PRO (Carestream Health Inc., Rochester, NY, USA).

### 2.10. Statistical Analysis

Data are expressed as the mean ± SEM. Statistical significance was assessed by Student's t-test or one-way analysis of variance followed by Tukey's post hoc test. All statistic analyses were performed using Prism GraphPad 5.0 (GraphPad Software Inc., La Jolla, CA, USA). P<0.05 was considered to indicate a statistically significant difference.

## 3. Results

### 3.1. IPostC Attenuates I/R Injury in the Heart via Autophagy and eNOS-Mediated Signaling

**Figure 1:**
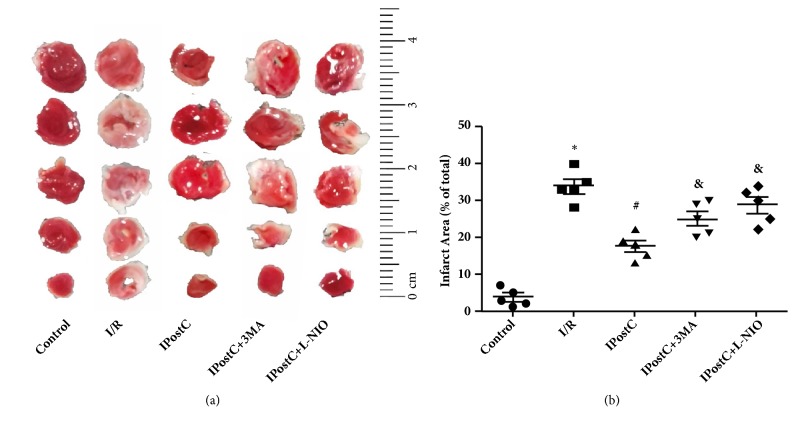
IPostC decreased the myocardial infarct size induced by I/R injury. Infarct size was measured following 2 h of reperfusion. I/R significantly increased myocardial infarct size and was decreased by IPostC. The protective effect of IPostC was abolished by autophagy inhibitor 3-MA or eNOS inhibitor L-NIO (n=5 per group). ^*∗*^P < 0.05 versus Control; ^#^P < 0.05 versus I/R; ^&^P < 0.05 versus IPostC.

**Figure 2:**
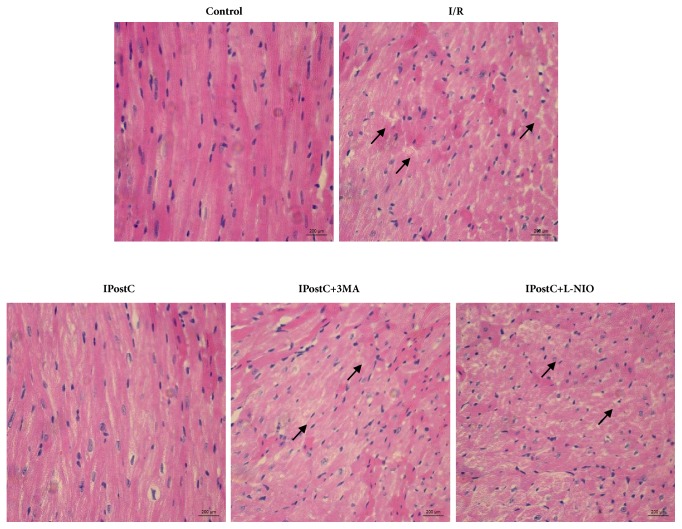
IPostC decreased the structural damage induced by I/R injury. Histological images of mice hearts following 2 h of reperfusion illustrate a grossly distorted structure, nuclear vacuolation, interstitial edema, and the appearance of a frequent contraction band. These structures were largely decreased in IPostC hearts. An autophagy inhibitor 3-MA or eNOS inhibitor L-NIO abolished the protective effect of IPostC. n=6 per group.

Firstly, the protective effect of IPostC on an I/R injured heart was verified, and the role of autophagy and eNOS in IPostC was investigated. As shown in Figure 1, I/R injury significantly increased the infarct size, when compared with the control group (33.8±1.9 versus 3.6±1.1%; P<0.05), whereas IPostC decreased the I/R-induced infarct size in the heart (17.4±1.6% versus 33.8±1.9%; P<0.05). The protective effects of IPostC were suppressed by the autophagy inhibitor 3-MA (25.1±2.1 versus 17.4±1.6%; P<0.05) or the selective eNOS inhibitor L-NIO (28.6±2.2 versus 17.4±1.6%; P<0.05). Secondly, myocardial structure was evaluated by H&E staining. As shown in Figure 2, murine hearts were collected after 2 h of reperfusion in order to obtain representative histological images. Evident ischemic changes, including nuclear vacuolation, interstitial edema, a grossly distorted structure, and a frequent contraction band, appeared in an I/R injured heart. IPostC markedly decreased the structural damage induced by I/R injury, and the autophagy inhibitor 3-MA or eNOS inhibitor L-NIO abolished IPostC protection. These results suggest that eNOS and autophagy participate in the protective effect of IPostC on the I/R injured hearts of mice.

### 3.2. IPostC Increases Autophagy via eNOS-Mediated Signaling in an I/R Injured Heart

**Figure 3:**
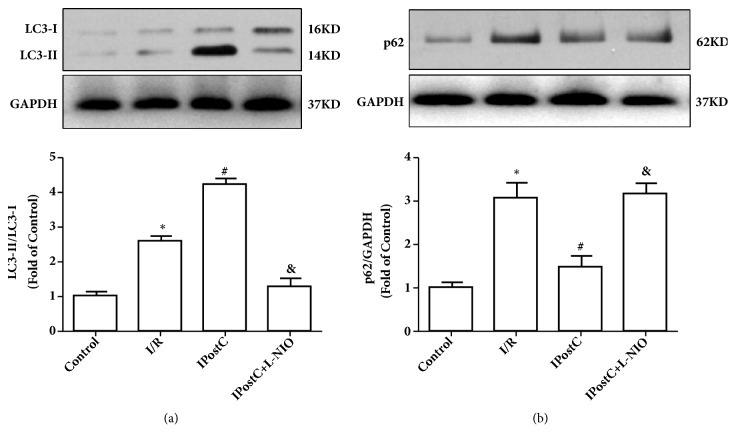
IPostC increased autophagy via eNOS-mediated signaling and promoted autophagy via eNOS in I/R injured hearts. (a) The expression of LC3 in the myocardium at 30 min of reperfusion. IPostC increased the ratio of LC3-II/LC3-I compared to I/R group, which was abolished by eNOS inhibitor L-NIO. (b) IPostC decreased p62 expression compared with the I/R group, which was abolished by eNOS inhibitor L-NIO. n=6 per group. ^*∗*^P < 0.05 versus Control; ^#^P < 0.05 versus I/R; ^&^P < 0.05 versus IPostC.

It was next investigated whether IPostC promoted autophagy via the eNOS-mediated pathway, and the expression of autophagy indicators LC3 and p62 in the myocardium was measured. As demonstrated in Figure 3, IPostC significantly increased the ratio of LC3-II/LC3-I and the expression of p62 compared with I/R. However, the eNOS inhibitor L-NIO suppressed the effect of IPostC. These data demonstrate that IPostC induces autophagy by activating the eNOS-mediated pathway.

### 3.3. AMPK/eNOS-Mediated Signaling Pathway Regulates the Effect of IPostC on Autophagy

**Figure 4:**
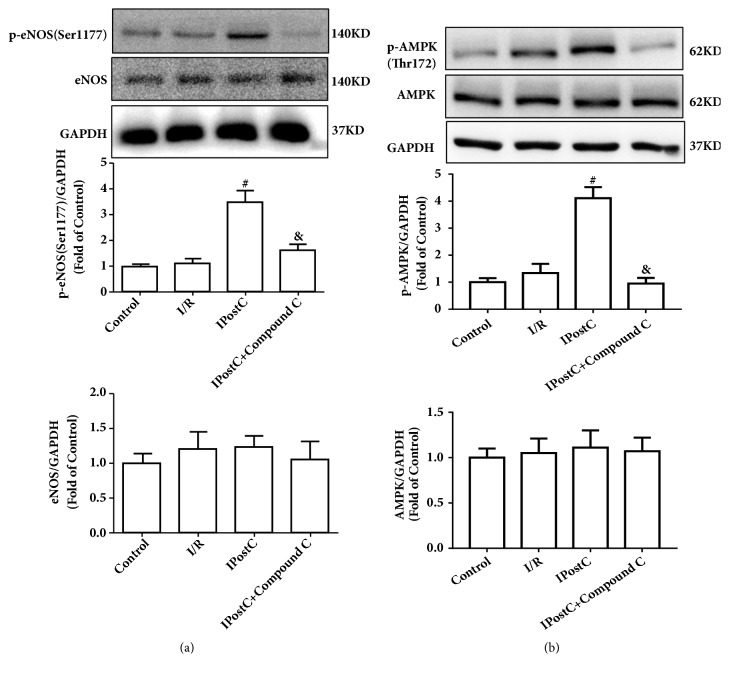
IPostC increased AMPK and eNOS phosphorylation in the myocardium. (a) IPostC increased the expression of p-eNOS^Ser1177^ compared with the I/R group in the hearts of mice, which was suppressed by the AMPK inhibitor Compound C. (b) IPostC increased the expression of p-AMPK^Thr172^ compared with the I/R group, and Compound C decreased the phosphorylation of AMPK in the heart of mice. n=6 per group. ^#^P < 0.05 versus I/R; ^&^P < 0.05 versus IPostC.

To further investigate the role of AMPK in IPostC-induced autophagy, the AMPK inhibitor Compound C was used and the expression of p-AMPK^Thr172^ and p-eNOS^Ser1177^ was measured. As illustrated in Figure 4, IPostC significantly increased the expression of p-eNOS^Ser1177^ compared with the I/R group, which was suppressed by the AMPK inhibitor Compound C. Additionally, IPostC markedly increased AMPK phosphorylation, and Compound C decreased the phosphorylation of AMPK. These data indicate that the activating effects of IPostC on autophagy are partially via AMPK/eNOS-mediated signaling pathways.

### 3.4. HPostC Protects H/R Injured H9c2 Cells via Activating Autophagy and eNOS-Mediated Signaling in Vitro

**Figure 5:**
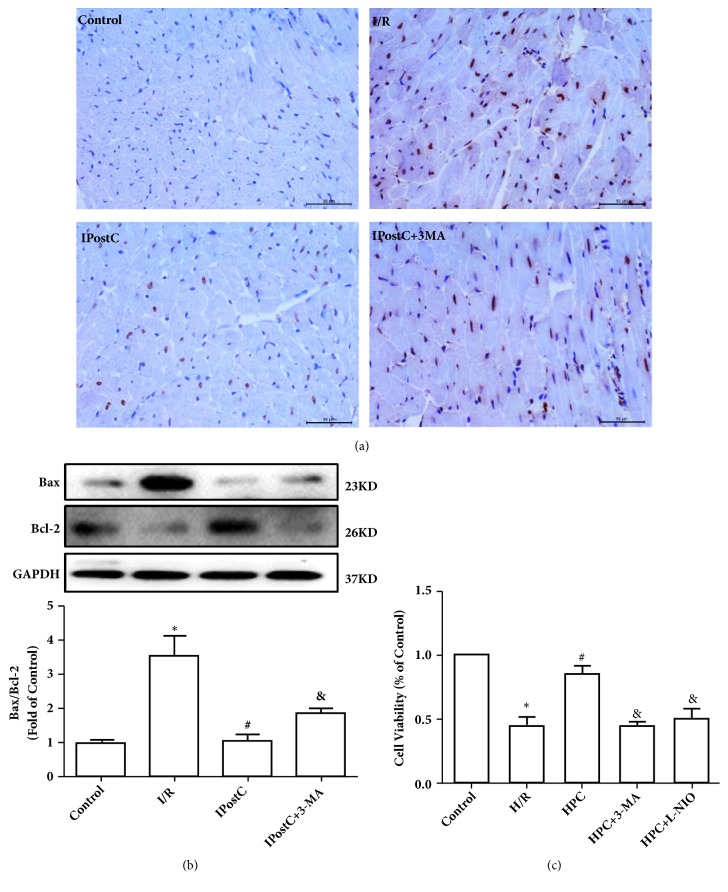
Autophagy and eNOS were involved in the protective effect of HPC against H/R injury in H9c2 cells. (a) Tunnel staining showed that IPostC protected against the apoptosis of myocardium induced by I/R injury, which was abolished by autophagy inhibitor 3-MA. n=3. (b) IR injury significantly increased the ratio of Bax/Bcl-2, which was reduced by IPostC. 3-MA abolished the protection effect of IPostC. n=6. (c) HPC decreased the apoptosis of H9c2 cells induced by H/R injury compared with the control group using the CCK-8 cell viability assay. HPC increased the cells proliferation against H/R injury, which was abolished by autophagy inhibitor 3-MA or the eNOS inhibitor L-NIO, n=3 per group. ^*∗*^P < 0.05 versus Control; ^#^P < 0.05 versus H/R; ^&^P < 0.05 versus HPostC.

To further verify our hypothesis that IPostC protects the I/R injured heart via activating autophagy, the apoptosis and viability of H9c2 cells were measured. As Figure 5(a) demonstrates, IPostC protected against the apoptosis of myocardium induced by I/R injury, which was abolished by autophagy inhibitor 3-MA. As shown in Figure 5(b), IR injury significantly increased the ratio of Bax/Bcl-2, which was reduced by IPostC. 3-MA abolished the protection effect of IPostC. As shown in Figure 5(c), HPostC decreased the cell apoptosis and activity induced by H/R. However, the autophagy inhibitor 3-MA or eNOS inhibitor L-NIO abolished the protection of HPostC. These data demonstrated that HPostC exerted its cardioprotective effects partially via activating autophagy and the eNOS-mediated pathway.

### 3.5. AMPK/eNOS-Mediated Autophagy Was Involved in HPostC Cardioprotection against H/R Injury in Vitro

**Figure 6:**
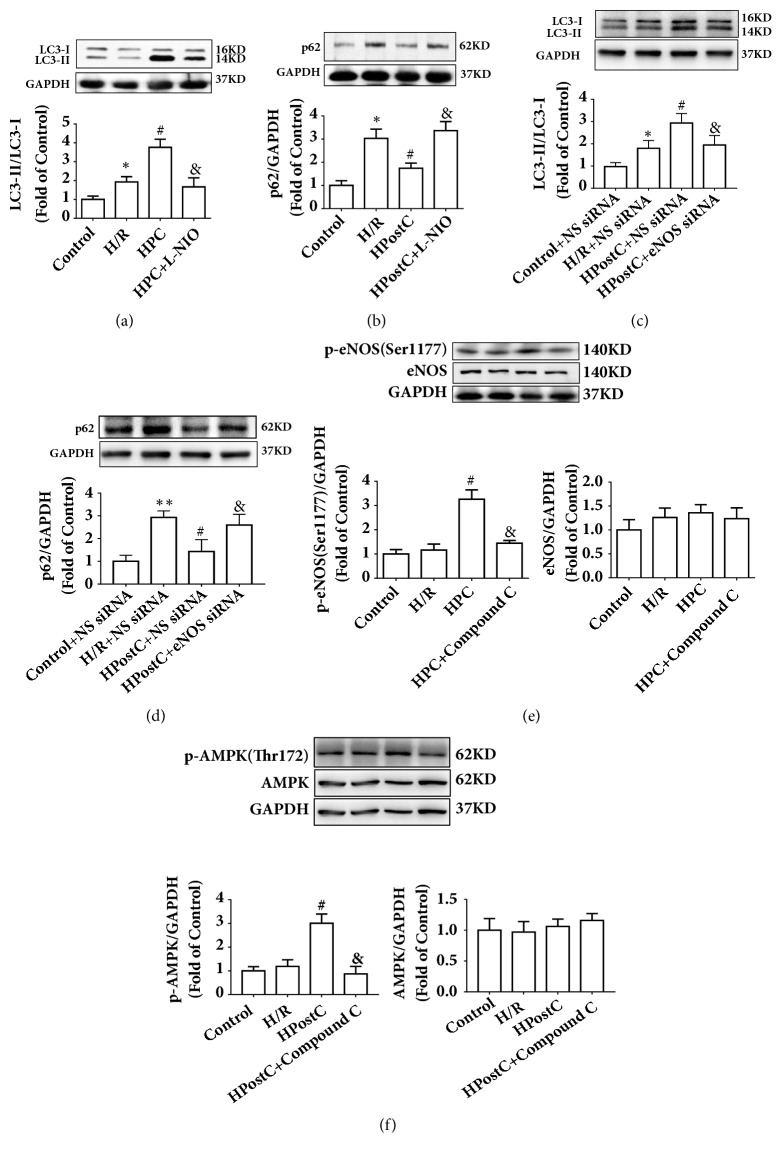
HPostC attenuated H9c2 cells H/R injury via activating AMPK/eNOS-mediated autophagy. (a), (b) HPostC increased the ratio of LC3-II/LC3-I and decreased p62 expression, which was abolished by the eNOS inhibitor L-NIO. (c), (d) eNOS siRNA abolished the effects of HPostC on the ratio of LC3-II/LC3-I and p62 expression. (e) HPostC increased the phosphorylation of eNOS. The AMPK inhibitor Compound C abolished the effect of HPostC on eNOS. (f) HPostC increased the expression of p-ANPK compared to H/R group, and Compound C decreased the phosphorylation of AMPK. n=3 per group. ^*∗*^P < 0.05, ^*∗∗*^P < 0.01 versus Control; ^#^P < 0.05 versus H/R; ^&^P < 0.05 versus HPostC.

The mechanism of HPostC cardioprotection observed* in vivo* was further verified in H9c2 cells* in vitro*. As shown in Figures 6(a) and 6(b), HPostC significantly increased the ratio of LC3-II/LC3-I and downregulated the expression of p62, which was also abolished by the eNOS inhibitor L-NIO. eNOS siRNA was also used to knockout gene of eNOS. As shown in Figures 6(c) and 6(d), eNOS knockout increased the ratio of LC3-II/LC3-I and decreased the expression of p62. In addition, the phosphorylation of eNOS was increased by HPostC, which was suppressed by the AMPK inhibitor Compound C (Figure 6(e)). When compared with the H/R group, AMPK phosphorylation was markedly increased by HPostC, which was decreased by Compound C (Figure 6(f)). These data suggest that HPostC activates autophagy via an AMPK/eNOS-mediated signaling pathway in H9c2 cells* in vitro*.

## 4. Discussion

The present study aimed to elucidate the mechanism of the protective effect of IPostC on a heart that has undergone I/R injury. The major findings were as follows: (i) autophagy and eNOS are involved in the protective effect of IPostC in an I/R injured heart; (ii) IPostC activates autophagy via eNOS-mediated signaling in an I/R injured heart; (iii) the AMPK/eNOS signaling pathway participates in activating IPostC in autophagy. Taken together, these findings demonstrate for the first time that IPostC attenuates I/R injury in the heart via AMPK/eNOS-mediated autophagy.

It is imperative to identify an effective treatment for I/R injury due to its high morbidity and mortality rates. Multiple studies have reported that ischemic preconditioning (IPre) has a protective effect in I/R injured hearts. However, due to the unpredictability of ischemia in the heart, IPre is hard to implement in the heart prior to ischemia, which limits the clinical applications of IPre. Therefore, more effective strategies are needed, such as IPostC or remote IPostC. IPostC, which is conducted within the first minutes of reperfusion, has been demonstrated to be an effective therapeutic modality on an I/R injured heart (25). Due to the confirmed therapeutic effects, it is essential to improve the mechanism of IPostC. In the present study, the cardioprotective effects of IPostC against I/R injury in mice were observed, and IPostC was demonstrated to reduce the size of infarction and improve the structure of the myocardium.

Autophagy, a key point in maintaining cellular homeostasis via removing the damaged organelles and proteins [29], has long been confirmed to occur in various tissues and organs, including cardiac tissue. A previous study reported that upregulating the expression of several genes associated with autophagy may decrease the apoptosis of cardiomyocytes in the chronically ischemic myocardium [30]. An additional study demonstrated that ischemic or pharmacological preconditioning activates autophagy and exhibits a protective function on ischemia heart [31, 32]. In the present study, it was demonstrated that autophagy participated in the cardioprotective effects of IPostC, when an autophagy inhibitor abolished the protective effects of IPostC. LC3-II, which is bound to the cell membrane in mature autophagosomes, is released upon fusion with the lysosome [32] and is used as a marker for the detection of autophagy. Additionally, p62 connects the LC3 protein and its ubiquitination substrates to mediate the clearance of proteins via autophagy [33]. The expression of p62 in cells directly reflects the level of autophagy completion [34]; therefore, the expression of LC3-II and p62 is measured in order to monitor the autophagic flow. The results of the present study demonstrated that the ratio of LC3-II/LC3-I was increased by I/R injury, indicating that the initial autophagosome formation was increased by I/R. However, p62 expression was also increased in the I/R group, indicating that there are problems in autophagy clearance and autophagic flux could not proceed smoothly. IPostC increased the ratio of LC3-II/LC3-I and decreased p62 expression compared with the I/R group, indicating that IPostC activates autophagy to attenuate I/R injury. Similar results were observed in H9c2 cells* in vitro*.

AMPK is widely expressed in the brain, heart, ovary, and uterus and in various other tissues in humans and mammals. AMPK is an important kinase to be considered as a ‘fuel gauge' for cellular energy levels [35]. AMPK is also a key molecule for the initiation of autophagy [36]. Previous studies have reported that AMPK-mTORC1 signaling is involved in the activation of autophagy by AMPK in cardiac ischemia [37, 38]. An additional study also revealed that AMPK enables the initiation of autophagy through phosphorylating and activating Unc-5-like autophagy activating kinase 1 directly [39]. The association between AMPK and autophagy following ischemia in the heart was further verified by identifying that the activation of autophagy in the early stages of ischemia followed the phosphorylation of AMPK, which was decreased by AMPK inhibition [40, 41]. A number of studies have reported that IPostC significantly increases AMPK phosphorylation and attenuates I/R injury in the heart. Therefore, AMPK-mediated autophagy may be involved in IPostC.

The serine residue 1177 of eNOS is a substrate for AMPK [42]. eNOS^Ser1177^ phosphorylation leads to the activation of eNOS and production of nitric oxide [43, 44]. AMPK/eNOS-mediated signaling has been confirmed to accelerate angiogenesis, particularly under the condition of ischemic stress [45, 46]. For the relationship between AMPK and eNOS, studies indicate that AMPK activation could directly phosphorylate eNOS in ECs and contribute to endothelial function [47, 48]. AMPK/eNOS-mediated signaling also participates in the vascular protection effect of ghrelin in ischemic myocardium of diabetic rats [49]. Therefore, AMPK/eNOS-mediated signaling may participate in IPostC-induced autophagy. In the present study, the role of AMPK/eNOS-mediated signaling in IPostC-induced autophagy was examined. The eNOS inhibitor blocked the cardioprotective effects of IPostC, decreased the LC3-II/LC3-I ratio induced by IPostC, and increased p62 expression in the heart of mice. These results indicated that eNOS-mediated autophagy served a role in IPostC. In addition, IPostC further increased AMPK phosphorylation when compared with I/R injury. The phosphorylation of eNOS was also increased by IPostC; however, it was abolished by AMPK inhibition. These results demonstrated that eNOS-mediated autophagy was AMPK-dependent and the AMPK/eNOS signaling pathway was involved in IPostC-induced autophagy. Similar results were observed in H9c2 cells* in vitro*.

Treatments in basic medicine for ameliorating I/R injury in the hearts of mice not only include ischemia postconditioning, remote IPostC, and pharmacological postconditioning, but also include recently the popular technology stem cell transplantation; for example, we could design and synthesize a novel iridium(III)-based probe 1 for discriminating stem cell lines from normal cell lines referring to other study, to track the trajectory of the stem cells. In this study, the autophagy-induced mechanism of AMPK/eNOS-mediated signaling, which participates in the protective effect of IPostC, may also have potential applications for the treatment of additional ailments. Future studies should aim to completely elucidate the mechanism of the presently available therapies and expand the understanding of the pathogenesis of I/R injury, in order to improve clinical treatment of the disease.

## 5. Conclusion

To the best of our knowledge, the present study is the first to report that eNOS serves an important role in autophagy induced by IPostC for attenuating the effects of an injury obtained by I/R. These findings further elucidate the mechanism of IPostC in protecting against I/R injury.

## Data Availability

The data used to support the findings of this study are available from the corresponding author upon request.
